# Driving Factors behind Energy-Related Carbon Emissions in the U.S. Road Transport Sector: A Decomposition Analysis

**DOI:** 10.3390/ijerph19042321

**Published:** 2022-02-17

**Authors:** Rui Jiang, Peng Wu, Chengke Wu

**Affiliations:** 1 Shenzhen Institute of Advanced Technology, Chinese Academy of Sciences, Shenzhen 518055, China; rui.jiang@siat.ac.cn (R.J.); ck.wu@siat.ac.cn (C.W.); 2 School of Design and the Built Environment, Curtin University, Perth 6102, Australia

**Keywords:** carbon emissions, carbon neutrality, renewable energy, electric vehicles

## Abstract

The U.S. is the second largest contributor to carbon emissions in the world, with its road transport sector being one of the most significant emission sources. However, few studies have been conducted on factors influencing the emissions changes for the U.S. from the perspective of passenger and freight transport. This study aimed to evaluate the carbon emissions from the U.S. road passenger and freight transport sectors, using a Logarithmic Mean Divisia Index approach. Emissions from 2008 to 2017 in the U.S. road transport sector were analysed and key findings include: (1) energy intensity and passenger transport intensity are critical for reducing emissions from road passenger transport, and transport structure change is causing a shift in emissions between different passenger transport modes; and (2) the most effective strategies to reduce carbon emissions in the road freight transport sector are to improve energy intensity and reduce freight transport intensity. Several policy recommendations regarding reducing energy and transport intensity are proposed. The results and policy recommendations are expected to provide useful references for policy makers to form carbon emissions reduction strategies for the road transport sector.

## 1. Introduction

Under the Paris Agreement, the U.S. government has committed to a 26–28% reduction target relative to 2005 emissions by 2025, and this target was updated to a 50–52% reduction in 2030 by President Biden in April 2021 [1,2]. The U.S. transport sector is one of the largest sources of energy consumption and greenhouse gas (GHG) emissions [3]. The U.S. transport sector contributed 28.2% of national GHG emissions, making it the largest contributor among all sectors in 2018 [4]. In addition, the transport sector accounted for 69% of the petroleum consumption in 2019, thus having a significant impact on the environment in terms of emissions [5]. In the transport sector, road transport is considered to be the biggest contributor to energy consumption and GHG emissions. Light, medium, and heavy vehicles accounted for more than 70% of energy consumption in the U.S. transport sector in 2019 and nearly 82% of GHG emissions in this sector in 2018 [6,7]. Given the significant impact of road transport on the sustainable performance of the entire transport sector, the U.S. Department of Transportation and the U.S. Environmental Protection Agency (EPA) are taking actions to gradually reduce carbon emissions. These actions include setting up new emissions standards and encouraging the use of renewable fuels [8].

It should be noted that the establishment of relevant policies to achieve emissions reduction relies on an accurate estimation of emissions and the identification of the influencing factors that have led to previous changes in emissions [9]. As such, many studies have been conducted to address this issue. For example, Ref. [10] analysed the CO_2_ emissions from the transport sector in China and found that the economic activities per capita and modal shifting in transport are the two biggest factors causing a significant increase in CO_2_ emissions. Studies have also been conducted in the road transport sector to identify the influencing factors of carbon emissions in this specific sector. For example, Ref. [11] decomposed the carbon emissions of Germany, Japan, South Korea, and Taiwan into five factors, which are the emission coefficient, fuel intensity, vehicle ownership, population intensity, and economic growth. It was found that although economic growth and the increase in vehicle ownership contribute significantly to the increase in carbon emissions, population intensity (i.e., the population growth per unit GDP) helps reduce the carbon emissions in the same period. Similarly, Ref. [12] developed a prediction model of carbon emissions from the road transport sector in China for 2050 and analysed the contributing factors that have influenced the carbon emissions level.

Several limitations of previous studies have been noticed. The road transport sector includes passenger transport and freight transport activities, which are usually differently measured. Correspondingly, the prediction of the future trend of emissions in the road transport sector should also be based on separate considerations of passenger transport and freight transport. However, a previous study, i.e., Ref. [10], adopted a conversion coefficient to integrate passenger transport and freight transport through experience, which may lead to inaccurate estimation. In addition, as one of the largest contributors to global emissions, the U.S. road transport sector has rarely been targeted. Refs. [13,14] investigated the factors that affected the transport-related emissions in the U.S. It was found that the dominant factors driving the U.S. transport CO_2_ emissions growth in 1970–1991 were growing population, travelling propensity, and gross domestic product (GDP). However, none of the existing studies has specifically examined the U.S. road transport sector, leaving a significant research gap about examining the effectiveness of emissions reduction policies and strategies in the U.S.

This study therefore aimed to: (1) assess the CO_2_ emissions of the U.S. transport sector from 2008 to 2017; (2) identify the drivers of changes in CO_2_ emissions and quantify their contribution from the perspective of passenger and freight transport; and (3) propose strategies that can be adopted to ensure that the transport sector can achieve its emissions reduction target. The contribution of this study is two-fold: (1) this study identifies factors driving the changes in CO_2_ emissions of the separate road passenger and freight transport sectors for the U.S., which are not revealed in existing studies; and (2) the policy recommendations proposed based on the findings of this study may provide solid references for policy makers and can be widely applied to other countries. The following section presents a literature review on the influencing factors of the emissions from road transport and mainstream decomposition analysis methods. Section 3 elaborates the method for emissions calculation and decomposition. The results and discussion are presented in Section 4 and Section 5, respectively. Section 6 concludes this study and provides policy implications of this research.

## 2. Literature Review

### 2.1. Factors That Influence the Emissions from Road Transport

Road transport is a critical component within the transport sector to ensure the sector achieves its emissions reduction target. As such, a number of studies have been conducted to evaluate drivers of emissions from the transport sector. Table 1 lists some of the studies, origins, research targets and the influence factors.

These current studies on road transport emissions can be categorised into three groups in terms of research targets, including road transport, passenger transport and freight transport. The first group of studies focus on road transport as a whole sector. The most commonly used influence indicators include energy intensity, carbon intensity, population and economic growth. Although these studies have revealed some useful insights, the inherent characteristics and relevant factors of passenger transport and freight transport can be different. For example, their energy structure is different because freight transport often relies more heavily on diesel than petrol. In addition, as shown in Table 1, population is an important factor that affects passenger transport but does not heavily affect freight transport. Treating passenger and freight transport as a whole may result in biased decomposition results [17].

As such, a number of studies have been initiated to separate passenger and freight transport. For example, Ref. [15] was one of the first that separately investigated road passenger transport and considered the effect of population, transport distance per capita, and emissions intensity. Ref. [16] compared the decomposition results of passenger car emissions from Denmark and Greece by looking at six factors, namely, population, vehicle ownership per capita, travelling distance, car fuel type, engine size and engine technology. For freight transport, Ref. [17] carefully examined the emissions characteristics of road freight transport in China and decomposed the emissions into nine relevant factors, including carbon intensity, vehicle load, distance, industrialisation level, and economic growth. Similarly, Ref. [18] investigated the emissions of the freight transport sector of China and analysed the contribution of a series of factors, including emission factor, market concentration, road freight market share, industrialisation level, fuel intensity and economic growth. It can be concluded that the factors related to passenger and freight transport are quite different and should be investigated separately. Based on the previous studies, emission factor, transport structure, energy structure, energy intensity, passenger transport intensity and population were examined for passenger transport emissions in this study. To enable a comparison between passenger and freight transport, the factors selected for freight transport are similar to those of passenger transport while considering the uniqueness of the sector. Therefore, emission factor, energy structure, energy intensity, freight transport intensity and GDP were adopted for the analysis of freight transport emissions.

### 2.2. Decomposition Analysis Methods

Index decomposition analysis (IDA) and structural decomposition analysis (SDA) are the most widely used methods in decomposition analysis [21,22]. Table 2 summarises their application condition, advantages and disadvantages. Generally, the IDA method is more widely adopted than the SDA method [23]. In addition, because the IDA method is usually used to analyse energy consumption or energy-related emissions changes in a specific sector, it was considered more suitable for this study.

Several IDA approaches are available, commonly based on the Divisia index and Laspeyres index. For example, the Logarithmic Mean Divisia Index (LMDI) and Arithmetic Mean Divisia Index (AMDI) methods are associated with the Divisia index, and the Fisher ideal index, Shapley/Sun and Marshall–Edgeworth methods are linked to the Laspeyres index. Considering the theoretical foundation, adaptability (e.g., data demand and sources), user-friendliness and results interpretation issues, Ref. [26] conducted a comprehensive comparison of these approaches and provided recommendations. It was noted that the main difference between the two indices is that the Laspeyres index focuses on the percentage changes, whereas the Divisia index is based on logarithmic changes. Due to the symmetry and additivity of the logarithmic changes, the Divisia index is considered to be more scientific than the Laspeyres index [26,27]. In addition, compared to the AMDI method, the LMDI is recommended for general use mainly because it is easy to implement and understand and provides perfect decomposition results without a residual term [26,28].

## 3. Method

### 3.1. Evaluation of CO_2_ Emissions in Road Transport

#### 3.1.1. Data Collection

The CO_2_ emissions in road transport were calculated using two sources of data, including energy consumption and CO_2_ emissions coefficients. The energy consumption data in the U.S. were collected from the Transportation Energy Data Book. At the time of this research, the latest edition was Transportation Energy Data Book Edition 38. It includes energy sources and consumption of the transport sector in 2017 [29]. This study evaluates the CO_2_ emissions of road transport from 2008 to 2017 to ensure data consistency because of a change in the method for evaluating energy use by the Federal Highway Administration. The Transportation Energy Data Book publishes transport energy by mode (including light vehicles, buses and medium/heavy trucks) and energy type (including road transport-related petrol, diesel, liquefied petroleum gas, natural gas and electricity). In addition, the CO_2_ emissions coefficients published by the U.S. were retrieved from U.S. Energy Information Administration [30]. For the CO_2_ emissions coefficients of electricity generation, the State Electricity Profiles (under the summary section) of the U.S. refer to the U.S. Energy Information Administration [31].

#### 3.1.2. CO_2_ Emissions Evaluation

As five sources of energy, namely, petrol, diesel fuel, liquefied petroleum gas (LPG), natural gas and electricity, are included, the CO_2_ emissions from road transport are calculated by Equation (1).

(1) $$CO_{2,road}=\underset{i}{\sum }CO_{2,i}=\underset{i}{\sum }E_{i}\times CC_{i}$$

where:

*E_i_* represents the energy use from energy source *i*; and *CC_i_* refers to the CO_2_ emissions coefficients of energy source *i*.

### 3.2. The LMDI Decomposition Approach

The LMDI method was adopted to analyse the influencing factors affecting the changes in CO_2_ emissions of the road transport sector in the U.S. It should be noted that passenger transport and freight transport are reported differently because they are affected by different potential influencing factors. As such, two separate decomposition models were developed for passenger transport, including light vehicles (cars, light trucks, motorcycles) and buses, and freight transport (medium/heavy trucks). Minivans, sport utility vehicles and light pickup trucks are all included in light trucks.

Equation (2) is used for the decomposition analysis of passenger transport [32]:

(2) $$CO_{2,P}=\underset{ij}{\sum }{\left(CO_{2,P}\right)}_{ij}=\underset{ij}{\sum }\frac{{\left(CO_{2,P}\right)}_{ij}}{E_{P,\;ij}}\times \frac{E_{P,ij}}{E_{P,i}}\times \frac{E_{P,i}}{E_{P}}\times \frac{E_{P}}{V}\times \frac{V}{P}\times P=\sum_{ij}EF_{P,ij}\times TS_{P,ij}\times ES_{P,i}\times EI_{P}\times PI\times P\;\;$$

where:

$CO_{2,P}$ represents the CO_2_ emissions from passenger transport; $E_{P,\;ij}$ represents the energy use of source i in mode j in passenger transport; $E_{P,i}$ denotes the energy use of source i for passenger transport; $E_{P}$ refers to the total energy use of passenger transport; V is passenger transport service (measured by million passenger miles/kilometres); P is population. $EF_{P}$ represents emission factor; $TS_{P}$ refers to transport structure, i.e., the share of transport mode j in the passenger transport sector; $ES_{P}$ refers to energy structure, i.e., the share of energy source *i* in the passenger transport sector; $EI_{P}$ represents the energy intensity of the passenger transport sector; PI means the passenger transport intensity (measured by transport distance per capita).

The changes in CO_2_ emissions in the passenger transport sector can therefore be decomposed into the six factors of $EF_{P}$, $TS_{P}$, $ES_{P}$, $EI_{P}$, PI and P, using Equations (3)–(9) [32].

(3) $$\Delta CO_{2,P}=\Delta CO_{2,EF_{P}}+\Delta CO_{2,TS_{P}}+\Delta CO_{2,ES_{P}}+\Delta CO_{2,EI_{P}}+\Delta CO_{2,PI}+\Delta CO_{2,P}$$

(4) $$\Delta CO_{2,EF_{P}}=\sum_{ij}L\left(CO_{2,ij}^{T},CO_{2,ij}^{0}\right)\;ln\frac{EF_{P,ij}^{T}}{EF_{P,ij}^{0}}=\sum_{ij}\frac{CO_{2,ij}^{T}-CO_{2,ij}^{0}}{lnCO_{2,ij}^{T}-lnCO_{2,ij}^{0}}\;ln\frac{EF_{P,ij}^{T}}{EF_{P,ij}^{0}}$$

(5) $$\Delta CO_{2,TS_{P}}=\sum_{ij}L\left(CO_{2,ij}^{T},CO_{2,ij}^{0}\right)\;ln\frac{TS_{P,ij}^{T}}{TS_{P,ij}^{0}}=\sum_{ij}\frac{CO_{2,ij}^{T}-CO_{2,ij}^{0}}{lnCO_{2,ij}^{T}-lnCO_{2,ij}^{0}}\;ln\frac{TS_{P,ij}^{T}}{TS_{P,ij}^{0}}$$

(6) $$\Delta CO_{2,ES_{P}}=\sum_{ij}L\left(CO_{2,ij}^{T},CO_{2,ij}^{0}\right)\;ln\frac{ES_{P,i}^{T}}{ES_{P,i}^{0}}=\sum_{ij}\frac{CO_{2,ij}^{T}-CO_{2,ij}^{0}}{lnCO_{2,ij}^{T}-lnCO_{2,ij}^{0}}\;ln\frac{ES_{P,i}^{T}}{ES_{P,i}^{0}}$$

(7) $$\Delta CO_{2,EI_{P}}=\sum_{ij}L\left(CO_{2,ij}^{T},CO_{2,ij}^{0}\right)\;ln\frac{EI_{P}{}^{T}}{EI_{P}{}^{0}}=\sum_{ij}\frac{CO_{2,ij}^{T}-CO_{2,ij}^{0}}{lnCO_{2,ij}^{T}-lnCO_{2,ij}^{0}}ln\frac{EI_{P}{}^{T}}{EI_{P}{}^{0}}$$

(8) $$\Delta CO_{2,PI}=\sum_{ij}L\left(CO_{2,ij}^{T},CO_{2,ij}^{0}\right)\;ln\frac{PI^{T}}{PI^{0}}=\sum_{ij}\frac{CO_{2,ij}^{T}-CO_{2,ij}^{0}}{lnCO_{2,ij}^{T}-lnCO_{2,ij}^{0}}ln\frac{PI^{T}}{PI^{0}}$$

(9) $$\Delta CO_{2,P}=\sum_{ij}L\left(CO_{2,ij}^{T},CO_{2,ij}^{0}\right)\;ln\frac{P^{T}}{P^{0}}=\sum_{ij}\frac{CO_{2,ij}^{T}-CO_{2,ij}^{0}}{lnCO_{2,ij}^{T}-lnCO_{2,ij}^{0}}ln\frac{P^{T}}{P^{0}}$$

The decomposition model was also developed for the freight transport, as shown in Equation (10).

(10) $$\begin{array}{cc} CO_{2,F}=\underset{ij}{\sum }{\left(CO_{2,F}\right)}_{ij} & =\underset{ij}{\sum }\frac{{\left(CO_{2,F}\right)}_{ij}}{E_{F,\;i}}\times \frac{E_{F,i}}{E_{F}}\times \frac{E_{F}}{F}\times \frac{F}{G}\times G\\ & =\sum_{ij}EF_{F,ij}\times ES_{F,i}\times EI_{F}\times FI\times G \end{array}$$

where:

$CO_{2,F}$ represents the CO_2_ emissions from freight transport; $E_{F,i}$ denotes the energy use of source i in this sector; $E_{F}$ is the total energy use in freight transport; F refers to the total freight transport service (measured by million tonne miles/kilometres); G is the national GDP. $EF_{F}$ represents emission factor; $ES_{F}$ represents energy structure; $EI_{F}$ represents energy intensity; and FI is the freight transport intensity, i.e., the freight transport distance per GDP. The calculation is similar to Equations (3)–(10). As energy use for medium and heavy trucks is not separately counted, transport structure was not considered for the freight transport sector.

Data related to transport activities, including passenger transport and freight transport, were collected from Organisation for Economic Co-operation and Development (OECD) database [33,34]. The population and GDP data were retrieved from The World Bank [35].

## 4. Results

### 4.1. CO_2_ Emissions of the U.S. Road Transport Sector

**Figure 1:**
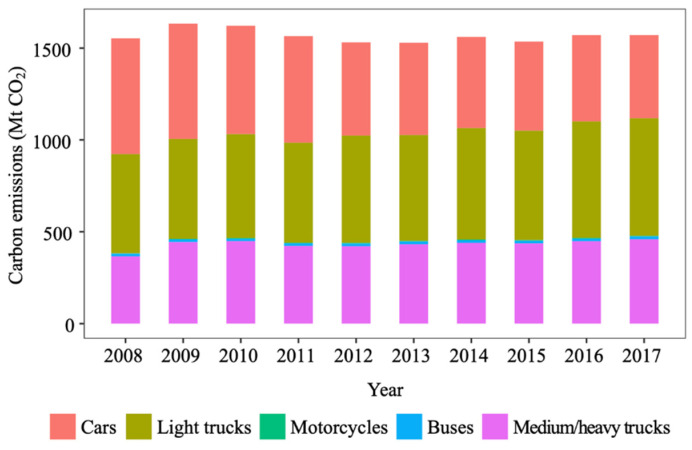
CO_2_ emissions of the U.S. road transport sector.

Figure 1 shows the annual CO_2_ emissions of the U.S. road transport sector from 2008 to 2017. The annual CO_2_ emissions grew slightly from 1.55 billion tonnes in 2008 to 1.57 billion tonnes in 2017. This number is consistent with a few reports on GHG emissions from the U.S. transport sector, e.g., [36]. It appears that road transport, representing 82% of the emissions from the U.S. transport sector [7], has contributed negatively to achieving the sector’s emissions target.

Passenger transport (cars, light trucks, motorcycles and buses) and freight transport (medium/heavy trucks) contribute an average of 72.5% and 27.5% to total road transport CO_2_ emissions, respectively. The annual CO_2_ emissions from passenger transport decreased from 1.19 billion tonnes in 2008 to 1.11 billion tonnes in 2017, which is a 6.7% decrease. Cars and light trucks are the two most important contributors. The contribution of cars to total road transport emissions decreased every year from 40.6% in 2008 to 28.8% in 2017, whereas the share of light trucks increased from 34.8% in 2008 to 40.7% in 2017. The share of motorcycles and buses was negligible. In addition, a general increasing trend can be identified in emissions from light trucks, motorcycles, and buses. The decrease in CO_2_ emissions in passenger transport can therefore be fully attributed to the reduced emissions of cars, which contributes a decrease of 0.18 billion tonnes of CO_2_ emissions. Comparatively, a general increase trend is identified in freight transport emissions. The annual CO_2_ emissions value of medium/heavy trucks increased from 0.37 billion tonnes in 2008 to 0.46 billion tonnes in 2017; a 24.3% increase. It should also be noted that, in 2009, there was a sharp increase in the carbon emissions from the freight transport sector. Due to this sharp increase, 2009 was also the year with the highest quantity of carbon emissions from the freight transport sector in the 10-year analysis period.

### 4.2. Decomposition Analysis of the U.S. Road Transport Sector

#### 4.2.1. The U.S. Road Passenger Transport Sector

**Figure 2:**
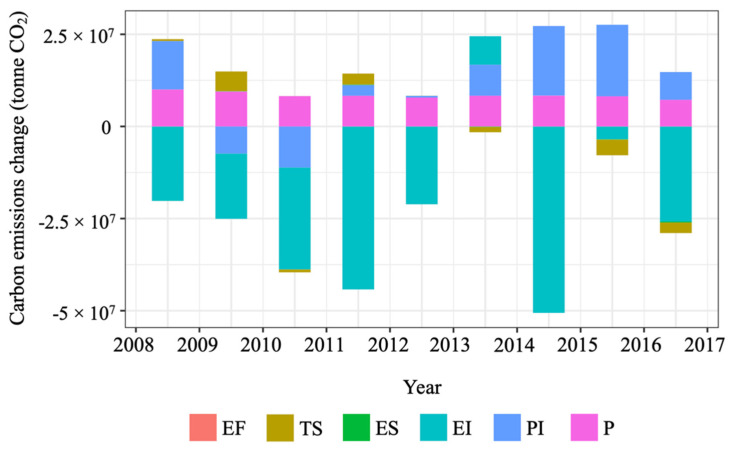
Decomposition results for the U.S. CO_2_ emissions of road passenger transport.

Table 3 and Figure 2 show the results for decomposition analysis of CO_2_ emissions in the U.S. road passenger transport sector. The aggregated changes in CO_2_ emissions in this sector from 2008 to 2017 were −75.80 million tonnes. Energy intensity is a key driver of such changes, causing a change of −203.06 million tonnes of CO_2_. This indicates that improved energy intensity may be the most effective strategy to reduce CO_2_ emissions over the analysis period. Interestingly, although the total contribution of the transport structure is limited, the fuel share of cars and light trucks in total energy consumption was −138.94 and 136.77 million tonnes CO_2_, respectively. Population consistently contributed to the increase in CO_2_ emissions, valued at 75.89 million tonnes. Although passenger transport intensity has an aggregated positive impact of 52.30 million tonnes of CO_2_ emissions, its contribution was inconsistent from 2008 to 2017. As shown in Figure 2, the yearly contribution of passenger transport intensity to the emissions change was higher in certain years (e.g., 2015–2016).

#### Positive Factors

**Figure 3:**
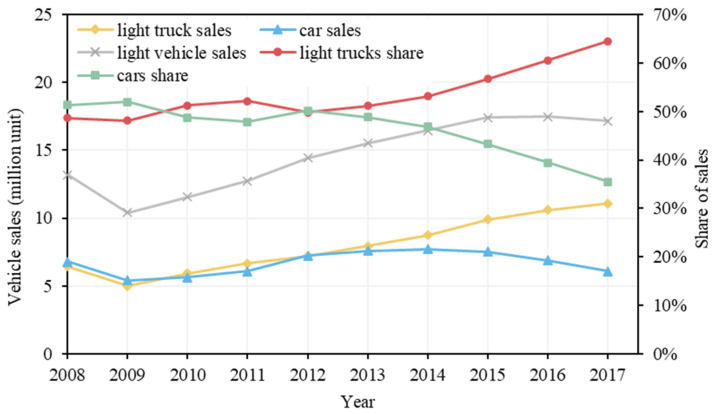
Vehicle sales in the U.S. from 2008 to 2017.

The fuel share of light trucks, population and passenger transport intensity are three notable factors that contributed positively to the changes in CO_2_ emissions. The fuel share of light trucks in total energy consumption in road passenger transport increased consistently from 45.5% in 2008 to 57.5% in 2017. This led to 136.77 million tonnes of CO_2_ emissions. Figure 3 shows that, in the recovery from the 2008–2009 financial crisis, vehicle purchases in the U.S. began climbing in 2010 [37]. The rate of light truck sales has a general increasing trend. With the decline in the petrol and diesel retail price [38], consumers’ preference for light trucks continued to increase from 2013 and peaked in 2017 at 64.5%. Given the dominant share of light trucks, lowering their emissions will be critical to achieving the emissions reduction target.

**Figure 4:**
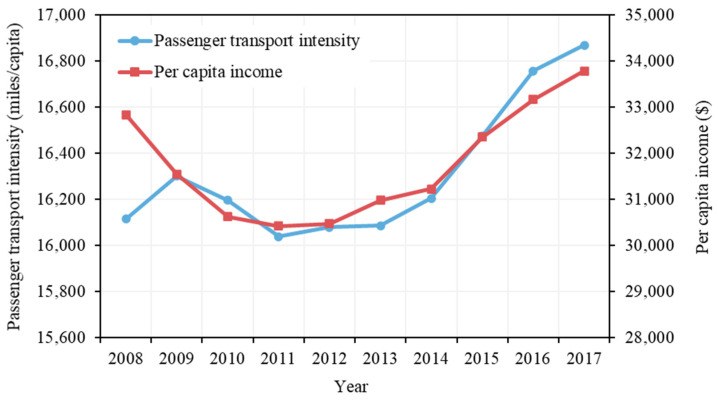
Passenger transport intensity and per capita income of the U.S. from 2008 to 2017.

The population in the U.S. increased steadily from 304.09 million in 2008 to 324.99 million in 2017, at an average annual rate of 0.76%. The increase in population led to 75.89 million tonnes of CO_2_ emissions during the 10 years. In addition, passenger transport intensity (kilometres/miles travelled per capita) contributed to 52.30 million tonnes of CO_2_ emissions from 2008 to 2017. Generally, passenger transport intensity increases as people get richer [39]. As shown in Figure 4, passenger transport intensity in the U.S. generally increased and the per capita income of the U.S. had a similar trend [40]. A sharp decrease is identified from 2009 to 2011, which may reflect the influence of the 2008 recession, as the per capita income of the U.S. reached its lowest level during the analysis period in 2011. Moreover, the transport intensity has increased since 2012, suggesting that the growth in passenger travelling distance has surpassed that of population. As average vehicle occupancy and driving distance per vehicle generally remain stable at about 1.67 passengers and 12,000 miles per vehicle, respectively [41,42], the increasing passenger travelling distance can be mainly attributed to an increase in the number of vehicles in use; that is, the group of people demanding a vehicle grew at a higher rate than that of the population.

#### Negative Factors

**Figure 5:**
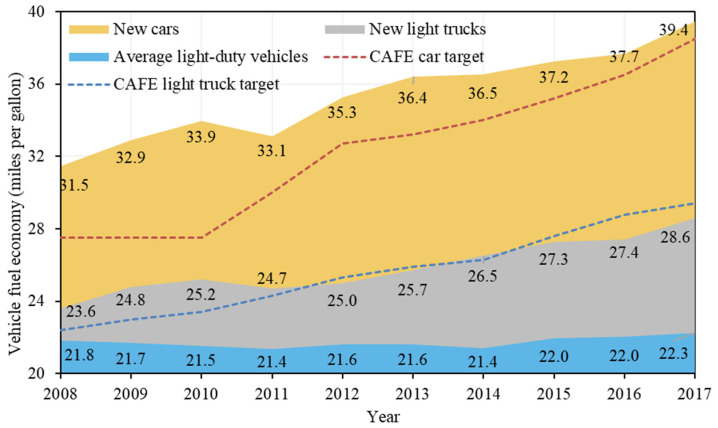
Fuel economy of light-duty vehicles in the U.S. from 2008 to 2017 (Source of data: [43]).

Energy intensity and the fuel share of cars are two notable factors that lowered the emissions. Energy intensity contributed −203.06 million tonnes of CO_2_ emissions of the U.S. road passenger transport sector from 2008 to 2017. Energy intensity is the energy use for transporting a passenger by 1 kilometre/mile and can be used to evaluate the fuel economy in road transport. Lower energy intensity indicates higher fuel economy. A decrease in fuel use and an increase in passenger transport service are both observed from 2008 to 2017, indicating a significant improvement in fuel economy occurred. Figure 5 shows that the energy economy of U.S. light vehicles, especially new vehicles, generally increased from 2008 to 2017. This indicates that the fuel economy and GHG standards set for cars and light trucks have worked effectively towards achieving the reduction target [42]. In the U.S., corporate average fuel economy (CAFE) standards released by the National Highway Traffic Safety Administration (NHTSA) and GHG emission standards enacted by the EPA are two sets of parallel standards for light-duty vehicles. The fuel economy standards were first established in 1975 and the GHG emissions standards first took effect for model year 2012, in harmony with the former standards. It is also worth noting that the standards are more stringently applied for cars than for light trucks. Considering the rising share of light truck purchases and fuel consumption, fuel economy requirements for light trucks should be raised.

The fuel share of cars in total passenger transport energy consumption decreased from 53.1% in 2008 to 40.7% in 2017, contributing to −138.94 million tonnes of CO_2_ emissions. This decrease is closely correlated to the increasing fuel share of light trucks because consumers are preferring light trucks over passenger cars. Therefore, the emissions simply shift between the two transport modes and the aggregated contribution of transport structure to emissions change is negligible.

#### 4.2.2. The U.S. Road Freight Transport Sector

As presented in Table 4, the CO_2_ emissions of the U.S. road freight transport sector increased by 92.41 million tonnes in total from 2008 to 2017. The most significant factors that contributed to the increase in emissions were GDP and energy intensity, which contributed 115.40 and 53.90 million tonnes of CO_2_, respectively, to the overall changes. On the contrary, freight transport intensity is a major driver of the decrease in CO_2_ emissions. This indicates that reducing freight transport intensity may be the most effective emissions reduction strategy in this sector. The contribution of the emission factor and energy structure is negligible.

**Figure 6:**
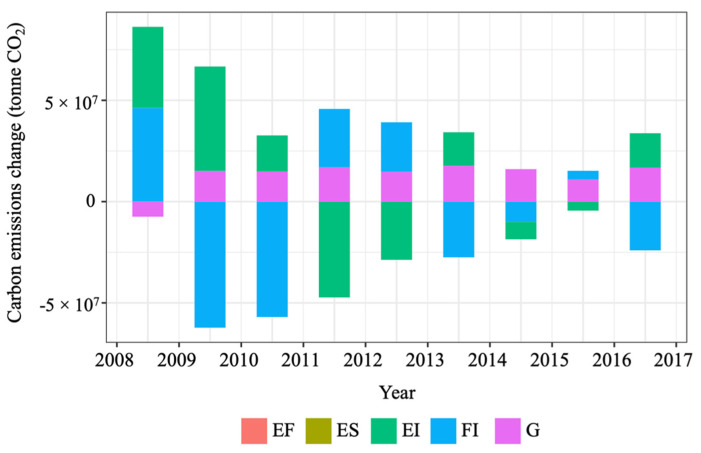
Decomposition results for the U.S. CO_2_ emissions in road freight transport.

Figure 6 shows the contribution of the five factors to the emissions changes on a yearly basis. In line with Figure 1, a sharp increase in CO_2_ emissions from 2008 to 2009 is identified. Freight transport intensity and energy intensity were the two most significant contributing factors to the increase. Their effects were 46.24 and 40.05 million tonnes, from 2008 to 2009, respectively. Figure 6 also shows that the effect of freight transport intensity and energy intensity on yearly emissions changes was significantly higher than other factors including GDP, especially in the early period from 2008 to 2013.

#### Positive Factors

GDP and energy intensity are two positive factors affecting the CO_2_ emissions changes in the U.S. road freight transport sector. GDP reflects the level of economic development of a country, and the GDP of the U.S. increased from USD 14,712.84 billion in 2008 to USD 19,485.39 billion in 2017; a 24.5% increase. The cumulative effect of such growth in GDP on the environment was 115.40 million tonnes of CO_2_ emissions.

**Figure 7:**
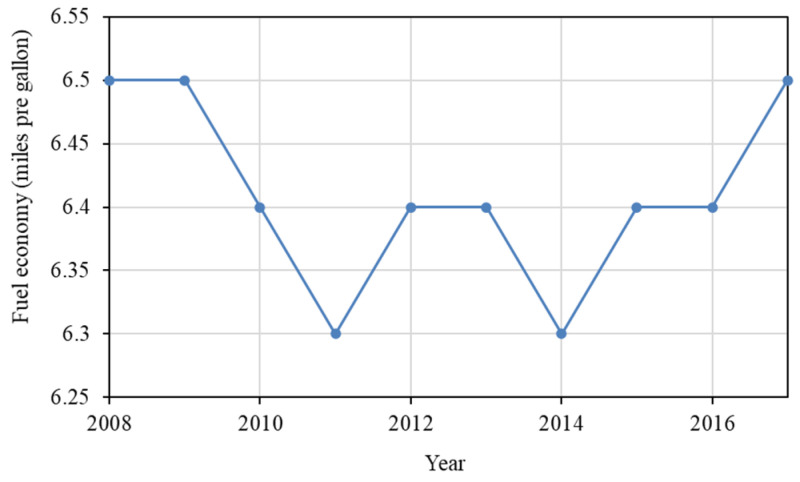
Fuel economy of medium- and heavy-duty vehicles in the U.S. from 2008 to 2017.

Energy intensity (an indicator for fuel economy) is another important factor that affects the changes in CO_2_ emissions of the U.S. freight transport sector, and contributed 53.90 million tonnes CO_2_. As shown in Figure 7, the annual fuel economy of medium- and heavy-duty vehicles in the U.S. experienced a down and up period, reaching its lowest value of 6.3 mpg in 2011 and 2014, and the peak value at 6.5 in 2008, 2009, and 2017 [44]. In 2011, the NHTSA and the EPA jointly established the first set of harmonised fuel economy and GHG emission standards (known as the Phase 1 standards) for medium- and heavy-duty vehicles. Although the standards took effect for model years 2014–2018, they were voluntary for model years 2014–2015 and become mandatory in 2016. The standards seem to be effective, as demonstrated in Figure 7.

#### Negative Factors

**Figure 8:**
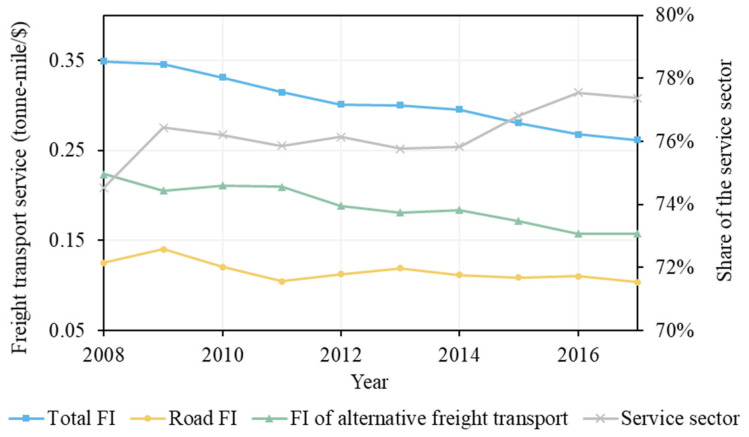
Freight intensity of the freight transport sector and share of service in the U.S. (FI: freight transport intensity).

Freight transport intensity indicates the transport demand per dollar of GDP that is created. Its aggregated effect was −76.89 million tonnes of CO_2_ emissions. According to [19], freight transport intensity can be impacted by the economic structure of a country because agriculture and industry need more transport than service. Figure 8 shows that although transport demand may shift between roads and alternative transport modes, the overall freight transport intensity in the U.S. decreased during 2008–2017 with the increasing share of the service sector. Road freight transport intensity also had a decreasing trend in this period, leading to the reduction in emissions. The reduced freight transport intensity can also be attributed to improved land use planning and development and/or local industry location [45].

## 5. Discussion

In previous studies that have examined the CO_2_ emissions of the road transport sector by looking at passenger transport and freight transport separately, it was found that passenger transport generally has higher impacts than freight transport. In this study, the contribution of passenger transport to the emissions from U.S. road transport was over 70%. It is interesting to find that the value of passenger service is usually 2.7 times the freight service value. With much higher fuel economy, passenger transport may have emitted lower emissions than freight transport. The large share of passenger transport in emissions can mainly be attributed to the low vehicle occupancy. Cars and light trucks transport more than 90% of the passengers and account for over 95% of passenger transport CO_2_ emissions. This explains why light vehicles are often specifically targeted for emissions reduction [45], and also highlights a need to rethink the importance of passenger transport structure in policy making (e.g., the share of public transport).

The overall changes in CO_2_ emissions in the passenger transport sector was −75.80 million tonnes from 2008 to 2017 in the U.S. Energy intensity was the most significant contributing factor driving the reduction in CO_2_ emissions in this sector, valued at −203.06 million tonnes of CO_2_. This indicates that improved energy efficiency may be one of the most efficient strategies towards emissions reduction. This finding supports [15] but disagrees with that of [46], who argued that the improvement in energy efficiency provided no significant energy and emissions savings for passenger cars. They drew such a conclusion because they included the impact of fuel efficiency improvement on the vehicle market and vehicle mileage in their consideration. In addition to population, passenger transport intensity (i.e., passenger miles travelled per capita) has been identified to be a dominant driver of the increase in CO_2_ emissions in U.S. road passenger transport. This agrees with the findings of [15], who identified per capita car driving distance to be a dominant factor driving the increase in CO_2_ emissions. Ref. [15] also argued that the emissions growth was caused by increasing per capita car trip distance and decreasing occupancy rate. Although the average vehicle occupancy rate and per capita car trip distance of the U.S. generally stayed unchanged during the analysis period [42,47], the main reason for its increasing passenger transport intensity can be attributed to the growth in vehicle ownership. Vehicle ownership has been identified to be the most influential factor by several studies, such as in [11,16], suggesting a demand for regulation at this point. For example, advocating the use of public transport or encouraging purchases of green vehicles such as electric vehicles instead of conventional vehicles should be helpful.

On the other hand, the aggregated changes in CO_2_ emissions in the freight transport sector were 92.41 million tonnes from 2008 to 2017 in the U.S. Although GDP has the highest aggregated effect, energy intensity and freight transport intensity (freight transport service per unit GDP) are the most significant drivers for the yearly changes in CO_2_ emissions. Energy intensity contributed 53.9 million tonnes CO_2_ in total and the positive effect echoes the findings of [19]. Ref. [18] found energy intensity to be a factor driving the decrease in CO_2_ emissions in China because of technological progress. In the U.S., emission standards for freight trucks have also shown the potential for reducing the energy intensity and emissions but they took effect in the latter stages of the studied period. Freight transport intensity contributes the most to the reduction in emissions in this sector. However, Ref. [19] identified freight transport intensity to be a positive factor on freight transport emissions in Tunisia, which is the opposite to the findings of this study. The reason may be that developed countries (e.g., the U.S.) often have a higher share of the service sector in their economic composition than developing countries (e.g., Tunisia) and the service sector relies less heavily on freight transport than agriculture and industry [48]. The effect of such differences can be further investigated in future studies by including developing countries into a more comprehensive comparison.

Since population and GDP are projected to constantly grow, reducing energy intensity and transport intensity are critical for achieving emissions reductions for both passenger and freight transport. Several policy recommendations can be made accordingly.

First, fuel economy and GHG emission standards for vehicles should be tightened. The 2021 CO_2_ emission targets for cars and light trucks in the EU are 95 g/km (153 g/mile) and 147 g/km (237 g/mile), respectively, and are renewed every five years [49]. For comparison, the 2021 targets for cars and light trucks in the U.S. are 172 g/mile and 249 g/mile [50]. Considering the increasing and dominant sales share of light trucks, the standards should be stringently applied to them. As the phase II standards for medium- and heavy trucks have been released for model year 2018 and newer, it is suggested to monitor the effect and make adjustment when necessary. Financial penalties (e.g., tax and rebate programs) for not complying these standards should also help.

Second, the electrification of vehicles should be accelerated. Pure battery electric vehicles were projected to save 41% of CO_2_ emissions compared to conventional gasoline internal combustion engine vehicles [51]. The CO_2_ emissions of electric vehicles entirely powered with electricity generated through renewable energy can be further reduced to 6 gCO_2_/km [52]. Compared to Norway, where electric vehicles (EVs) made up 75% of the new sales market in 2020, the sales share of electric vehicles in the U.S. was only 2% in the same year [53]. Well-designed carbon tax and/or carbon pricing programs can motivate manufacturers and dealers to provide and sell more green vehicles. As only twelve states are involved in carbon pricing programs [54], such market-based measures could be expanded. On the other hand, vehicle labelling with fuel information and financial incentives (e.g., tax exemption or feebate programs) can also be provided to encourage the purchase of such green vehicles instead of conventional vehicles. In addition, the EU recommended that 1 public charger should be available per 10 EVs at a 0.1 ratio in 2020 [53]. However, the ratio in the U.S. was 0.05, calling for more government investment into the public charging infrastructure [55]. Increasing the use of renewable energy in electricity generation is also critical; otherwise, the life cycle emissions of road transport will not be significantly reduced. It should also be noted that trucks weighting up to 18 tonnes used for local distribution can be powered with batteries. However, heavy trucks for long-distance distribution may need alternative strategies. As hydrogen fuel cell powered electric trucks are not yet ready for practical use, rail transport or cleaner fuels such as biodiesel should be encouraged. Low-carbon fuel standards or increasing the fuel price or tax for conventional fuels can be useful.

Finally, the effect of improved energy intensity can be easily offset by increased passenger/freight transport distances. The use of intelligent transport systems is an effective way of improving the transport efficiency as traffic congestion is an important source of emissions. Compact land use and development, achieved by locating communities, shopping, businesses and public transport stations close to each other, can also effectively reduce the driving distances by 20–30% compared to conventional development [45]. Similarly, better locating related industries can help reduce freight moving distances. In addition, signs can also be used to encourage alternative transport modes such as walking, cycling, public transport (for passenger transport) and rail (for passenger/freight transport).

Regarding the policy implementations, the three levels of government usually play different roles. The federal government often sponsors federal research and development on promising but challenging technologies such as clean hydrogen production, provides funding for financial incentives, and sets national mandates, regulations and carbon prices. State governments decide the allocation of the funds and may customise their own standards. Local governments are more engaged in the practical measures such as improving land use planning [56] and providing reliable public charging infrastructure.

## 6. Conclusions

This study evaluated the energy-related CO_2_ emissions of the U.S. road transport sector and decomposed these emissions to identify the factors that caused the changes in the emissions from 2008 to 2017. An LMDI approach was adopted for the decomposition analysis. Emissions from passenger and freight transport were separately analysed because they have different characteristics and influencing factors. Six factors were considered for the passenger transport sector, namely, emission factor, transport structure, energy structure, energy intensity, passenger transport intensity, and population. Among these factors, reduced energy intensity was found to be the most effective in reducing CO_2_ emissions. In addition to population, passenger transport intensity has a significant impact on the increase in CO_2_ emissions. The changes in CO_2_ emissions from the freight transport sector were decomposed into emission factor, energy structure, energy intensity, freight transport intensity, and GDP. Although the aggregated effect of GDP is high, energy intensity and freight transport intensity significantly drive the yearly changes in CO_2_ emissions from freight transport. Based on these findings, several policy recommendations are proposed to reduce energy and transport intensity to achieve emissions reduction targets.

There are also some limitations in this study. First, data for energy consumption are not reported in time, which may restrict the timeliness of the results. For example, the sales of electric vehicles in the U.S. have been climbing since 2017, which may have increased the electricity consumption. The GHG and fuel economy standards have also been extended to model year 2017–2025 vehicles. The COVID-19 pandemic is also likely to have impacts on transport intensity. The results could be improved if more data are available in the future. Nevertheless, as the pattern has been identified, the results are still illustrative for policy making. In addition, due to data limitations, the energy intensity of different fuel types was not separately investigated. Future research may further explore this point if more detailed data can be obtained, especially when electricity is more widely used. The change in vehicle engines may also be investigated in future studies. Furthermore, an estimation of the external costs of the recommended policies, such as promoting public passenger transport and railway freight transport, is also needed in future studies for more informed policy making.

## Figures and Tables

**Table 1:** A list of studies on investigating the influence factors of road transport emissions.

Year	Studies	Country	Targets	Influence Factors
2005	[15]	UK	Passenger	Emission coefficient; Fuel structure; Fuel intensity; Occupancy rate; Distance per person; Population.
2007	[11]	Taiwan, Germany, Japan and South Korea	Road Transport	Emission factor; Population intensity; Vehicle ownership; Fuel intensity; Economic growth.
2009	[16]	Greece and Denmark	Passenger	Ownership effect; Distance; Fuel mix; Car capacity change; Car engine change; Population.
2012	[17]	China	Freight	Carbon intensity; Vehicle technology level; Vehicle load; Transport company size; Number of transport companies; Distance; Relations between freight transport and industrialisation; Level of industrialisation; Economic growth.
2013	[18]	China	Freight	Emission factor; Market concentration; Road freight market share; Industrialisation level; Fuel intensity; Economic growth.
2014	[19]	Tunisia	Freight	Emission intensity; Energy intensity; Transport intensity.
2015	[12]	China	Road Transport	Share of vehicle types; Energy intensity of vehicle types; Emission intensity of vehicle types.
2017	[20]	Tunisia	Road Transport	Energy intensity; Economic development level; Urbanisation; Motorisation, Energy consumption.

**Table 2:** Advantages and disadvantages of the IDA and SDA methods.

Method	Application Condition	Advantages	Disadvantages
Index decomposition analysis (IDA)	Usually used to examine the driving factors of energy/energy-related emissions changes in a specific sector (e.g., the transport sector) [21,24,25].	(1) High flexibility in formulation and application [21]. (2) A large number of factors can be easily handled with the Logarithmic Mean Divisia Index (LMDI) method [21]. (3) Relatively low data requirement. Both data with high or low degree of sector disaggregation can be used [21].	(1) In SDA terminology, cannot deal with indirect effect [21]. (2) The application is limited in one-stage decomposition models [21].
Structural decomposition analysis (SDA)	Often employed by those comfortable with using input–output analysis [21,25].Primarily used to analyse the energy/emissions changes in the whole economy [21,24,25].	(1) Both direct and indirect effect is captured [21]. (2) Can include two-stage decomposition models [21].	(1) Relatively high data requirement [22]. (2) The application relies on the availability of input-output tables, limiting the flexibility [21].

**Table 3:** Results for the decomposition of the U.S. road passenger transport sector.

Year	CO_2_ Emissions Changes (Million Tonnes)										
	EF	TS					ES	EI	PI	P	Total
		Car	Light Truck	Motorcycle	Bus	Total					
2008–2009	0.00	−2.60	1.32	1.91	−0.12	0.52	−0.03	−20.21	13.16	10.01	3.46
2009–2010	0.00	−25.09	31.40	−0.30	−0.68	5.33	0.13	−17.72	−7.35	9.46	−10.16
2010–2011	0.00	4.89	−6.32	0.08	0.55	−0.80	0.02	−27.66	−11.14	8.22	−31.34
2011–2012	0.00	−54.88	57.40	0.53	−0.07	2.97	0.16	−44.23	2.90	8.31	−29.89
2012–2013	0.00	0.67	−0.81	−0.10	0.27	0.03	−0.01	−21.13	0.45	7.84	−12.82
2013–2014	0.00	−18.70	17.88	−0.13	−0.48	−1.43	−0.15	7.75	8.43	8.31	22.91
2014–2015	0.00	−1.09	0.98	−0.01	−0.05	−0.17	−0.02	−50.59	18.91	8.35	−23.52
2015–2016	0.00	−28.18	24.40	0.06	−0.52	−4.24	−0.15	−3.44	19.40	8.19	19.75
2016–2017	0.00	−13.95	10.52	−0.01	0.58	−2.87	−0.24	−25.83	7.55	7.21	−14.18
2008–2017	0.02	−138.94	136.77	2.03	−0.51	−0.65	−0.29	−203.06	52.30	75.89	−75.80

Note: EF—emission factor; TS—transport structure; ES—energy structure; EI—energy intensity; PI—passenger transport intensity; P—population.

**Table 4:** Results for the decomposition of the U.S. road freight transport sector.

Year	CO_2_ Emissions Changes (Million Tonnes)					
	EF	ES	EI	FI	G	Total
2008–2009	0.00	0.00	40.05	46.24	−7.44	78.86
2009–2010	0.00	0.00	51.55	−62.25	15.16	4.46
2010–2011	0.00	0.00	17.88	−56.97	14.81	−24.28
2011–2012	0.00	0.00	−47.32	28.80	16.94	−1.58
2012–2013	0.00	0.00	−28.75	24.52	14.64	10.41
2013–2014	0.00	0.00	16.61	−27.53	17.65	6.73
2014–2015	0.00	0.00	−8.65	−9.99	16.04	−2.60
2015–2016	0.00	0.00	−4.47	4.36	10.86	10.75
2016–2017	0.00	0.00	17.01	−24.08	16.74	9.68
2008–2017	0.00	0.00	53.90	−76.89	115.40	92.41

Note: EF—emission factor; ES—energy structure; EI—energy intensity; FI—freight transport intensity; G—GDP.

## Data Availability

The data presented in this study are available on request from the corresponding author.
